# Negative Pressure Wound Therapy in Infected Wound following Posterior Spinal Instrumentation using Simple Self-assembled System: A Case Report

**DOI:** 10.5704/MOJ.1407.004

**Published:** 2014-07

**Authors:** CW Chang, HZ Chan, SW Lim, EH Khoo, O Zulkiflee

**Affiliations:** Department of Orthopaedics, Penang General Hospital, George Town, Malaysia; Department of Orthopaedics, Penang General Hospital, George Town, Malaysia; Department of Orthopaedics, Penang General Hospital, George Town, Malaysia; Department of Orthopaedics, Penang General Hospital, George Town, Malaysia; Department of Orthopaedics, Penang General Hospital, George Town, Malaysia

## Abstract

Postoperative wound infection in an instrumented spine patient is often disastrous. Management includes implant removal leading to spine instability. Negative pressure wound therapy (NPWT) applied to the spine surgical wound is one of the wound care technique with successful results. We report a case of a man who sustained Chance fracture of Lumbar 1 (L1) vertebra treated with long segment posterior instrumentation, who unfortunately developed Extended-spectrum beta-lactamase (ESBL) positive E. coli infection one month after the operation. After careful debridement of the wound, the implant became exposed. Three cycles of NPWT were applied and the wound healed with granulation tissue completely covering the implant, and thus negating the need to remove the implant. In conclusion, the NPWT is a good alternative in postoperative wound management especially in an instrumented spine patient.

**Key Words:**

vacuum assisted closure, infection, instrumentation, spine

## Introduction

Postoperative wound infection is a severe complication after instrumented spine surgery. Treatment includes surgical debridement, antibiotic therapy, primary or secondary closure, or even removal of implant. The aim of the treatment is to eradicate infection without compromising the stability of the spine.

Negative pressure wound therapy (NPWT) has been in use to assist closure of problematic wounds. The application of this wound care technique in spine surgery produced good results with no complication. The implant can be left in situ and salvaged. In cases where infection is difficult to control, removal of implant can be delayed until the bone is united. This dressing technique creates a negative pressure microenvironment in the wound bed to promote neovascularisation and granulation tissue formation, and at the same time removes edematous fluid and reduces bacterial load.

We report a case of posterior instrumentation of lumbar spine complicated with deep surgical site infection. After aggressive debridement, the implant became exposed. However the wound healed with granulation tissues completely covering the implant after NPWT.

## CASE REPORT

**Fig. 1: Thoracolumbar radiograph of the patient after posterior instrumentation.**
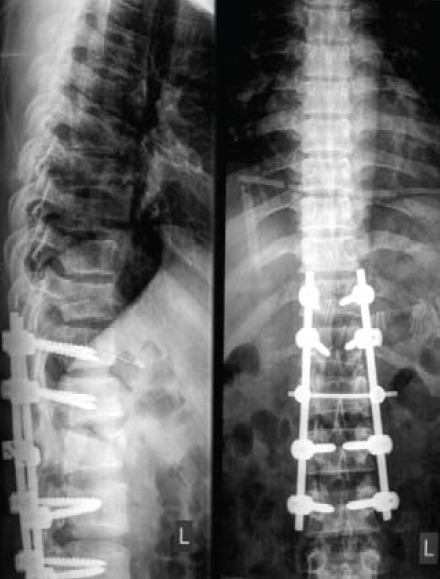


A 33 years old man sustained Lumbar 1 (L1) Chance fracture after a fall from 20 feet height. He underwent long segment posterior instrumentation from 11th thoracic to 3rd lumbar vertebrae (Figure 1). Unfortunately one month later, he developed deep surgical site infection. Wound debridement was done and he was started on ceftriaxone. The implant became exposed after the debridement (figure 2A).

**Fig. 3: Self-assembled NPWT system.**
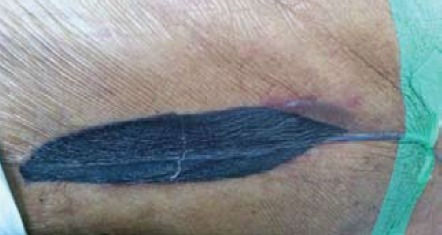


Post- debridement Day 1, the wound was healthy and free from necrotic debris. NPWT was applied using a simple self-assembled system. Two layers of open cell foam sponge were cut into the shape of the wound bed and suction tubing was laid in between the sponge layers. The wound was then sealed with occlusive film dressing (Figure 3). The tubing was connected to a suction canister with a pressure of 100-125 mmHg. After four days, the wound was clean and granulation tissue started growing. However the implant was still exposed. Culture and sensitivity grew Extended-spectrum beta-lactamase (ESBL) positive E. coli and antibiotic treatment was changed to imipenem and fusidic acid. Removal of implant was imminent but the patient refused.

Therefore, second cycle of NPWT was started. After four days, the wound was very clean and granulation tissue had covered the implant partially (Figure 2B). He was given a third cycle of NPWT for another four days. The wound was completely covered with granulation tissue and the implant was no longer visible after 12 days (figure 2C). He was discharged home after three weeks. On subsequent follow up of the patient as an outpatient, the wound had healed completely without sinus formation or pus discharge.

## Discussion

Despite today’s advances in sterile technique and antibiotic treatment, postoperative wound infection remains a major challenge to the surgeon. For spine surgery, the overall incidence of infection in instrumented cases has been shown to range from 3.6% to 10% ^1^.

The consequences of surgical site infection in an instrumented spine patient are usually devastating, resulting in increased mortality and morbidity, lengthened hospital stay, and increased hospital costs. In one group of patient with postoperative spinal wound infection, the estimated hospital cost increased by four times with an additional 58 days of hospital stay ^2^ . Besides surgical debridement and antibiotic treatment, wound care is an important and major factor in overall recovery. The NPWT used in this study consisted of a wall suction unit with a disposable canister and sponge with a suction tube. This self-assembled NPWT system is less expensive compared to the commercially available system. Conventional wound care using daily saline gauze dressing or colloid gel dressing is very labour intensive. The NPWT on the contrary requires only biweekly change.

In cases where infection is difficult to control, removal of implant prior to bone union may be necessary to eradicate the infection successfully. However it will result in complications such as non-union, spine instability or even irreversible neurological injury. Therefore a dressing method that is highly effective such as NPWT is required to salvage the implant.

In comparison with conventional dressing, NPWT is a semi-occlusive dressing that allows exposed implants to be covered up and stale collection to be drained out at the same time. The negative pressure microenvironment on the wound bed promotes blood flow, neovascularisation and fibroblastic proliferation, thus increasing the rate of granulation tissue formation. It also provides mechanical approximation of wound edges. Thus, overall healing potential is increased and allows faster wound coverage ^3^.

**Fig. 2: A – Post debridement day one, wound was clean but implant was exposed. B – After two cycles of NPWT, the wound was partially covered with healthy granulation tissue. C - The wound was completely covered with granulation tissue and the implant was no longer visible after 12 days.**
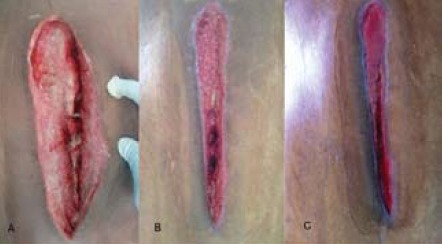


## Conclusion

Negative pressure wound therapy is an effective way to treat deep surgical site infection of the spine without removing the implants. Even in infection with resistant organism, we found that NPWT can be used with successful outcome. Thus, the use of NPWT may improve the overall care of the patient and help reduce the hospital cost.
